# Design of Nuclear Radiation Monitoring System in Floor Exploration Based on Deep Learning

**DOI:** 10.1155/2022/4351339

**Published:** 2022-05-21

**Authors:** Bochen Zong

**Affiliations:** School of Nuclear Science and Engineering, North China Electric Power University, Beijing 102206, China

## Abstract

Nuclear radiation environmental monitoring has become an important issue in floor surveys. From the perspective of regional environmental nuclear radiation monitoring, it is of great practical significance to establish a scientific and reliable wireless sensor monitoring network for timely and accurately grasping nuclear radiation status and ensuring nuclear safety. In this article, we design a regional environmental nuclear radiation monitoring system based on Zigbee wireless sensor network by using Zigbee wireless technology. First, the network topology suitable for nuclear radiation environment monitoring is designed. Second, the JN5121 module is selected as the core of the Zigbee wireless sensor to build the network hardware platform. Finally, the article focuses on the receiving mechanism, data transmission, coordination between network nodes, network structure, and dynamic network management, and carries on the software development to the Zigbee wireless environment monitoring network. The system can measure the data in real time, display the dose rate of *y* radiation effectively, and realize the functions of remote control, field control, security alarm, and environmental monitoring. It has good promotion and application value.

## 1. Introduction

In recent years, the frequent occurrence of the urban environment and land gas events makes the study of the release law of urban environment and land gas become the focus. For densely populated cities, artificial changes in the particularity of the surface structure will affect and change the natural release of Earth and air materials in the soil. It is easy to release in weak coverage and often causes anomalies. Zhou and Tao of Peking University put forward the concept of macro and micro effects of geog as based on the release characteristics of geog and its impact on the ecological environment and studied the phenomenon of long-distance transport of matter under the action of geog as and its significance in environmental research [1]. Du believes that upward emission of underground gases can cause at least 13 effects, revealing the environmental hazards of underground gases [2].

The floor survey and monitoring system needs to collect a lot of data, and it has a very high demand for wireless sensor networks [3]. Wireless sensor networks should not only track and monitor environmental changes but also not destroy the environment. At present, deep learning has become an innovative technology to solve the problems of floor exploration. In particular, Zigbee wireless sensor network is a research hotspot of scholars at home and abroad. Zigbee wireless sensor environmental monitoring system has a broad application prospect [4]. The research emphases include node device miniaturization technology and real-time operating system design based on the embedded device, protocol, and architecture construction of sensor network [5, 6].

Based on the above research, this topic from the floor to the exploration of the radiation monitoring perspective, using Zigbee wireless technology to design based on Zigbee wireless sensor network floor exploration of the radiation monitoring system, real-time measuring data, shows that the radiation dose rate effectively realizes the remote control, scene control, security alarm and environmental monitoring, and other functions. The system is of great significance to timely and accurately grasp the situation of nuclear radiation, nuclear accident emergency protection, ensure nuclear safety, and reduce the incidence of nuclear accidents.

## 2. System Requirement Analysis

To meet the needs of wireless ad hoc network floor survey and radiation environmental monitoring requirements, the first task is to determine the depth study adopts the wireless communication technology, according to the functional requirements of the whole system mainly from the wireless ad hoc network, collection and processing of various sensors, positioning and alarm functions, and combining with the characteristics of the regional environment, and determine various modules for the system. A wireless ad hoc network monitoring system is designed for floor investigation of nuclear radiation.

### 2.1. Demand Analysis of Key Technologies

At present, the network communication technologies used in wireless sensor networks mainly include wireless local area networks (Wi-Fi), ultra-wideband communication (UWB), Bluetooth, and Zigbee.

Wi-Fi has a long transmission distance and high speed, but it costs a lot and consumes a lot of energy. Wi-Fi technology relies on wireless network cards or access point devices applied to campus and corporate LANs and is suitable for indoor monitoring, not for outdoor monitoring needs [7].

Bluetooth has the advantages of low cost, low system power consumption, and small chip size. However, the biggest disadvantages of Bluetooth technology are high engineering cost, short communication distance (10 m), and easy eavesdropping of data communication, which puts Bluetooth technology at a disadvantage in the detection of the nuclear radiation environment.

UWB is characterized by fast transmission speed, strong anti-interference ability, very small transmitting power, and low electromagnetic radiation to the human body. That is because of these advantages that some people believe that it will become the mainstream technology of short-distance wireless communication in the future [8]. But the current working group on UWB standards has failed to come up with a final standard, and the technology still needs to be refined because there is no consensus.

Table 1 shows the existing mainstream wireless sensor network technologies.

As can be seen from the comparison in Table 1, Zigbee is selected mainly from the perspective of low power consumption (compared with Bluetooth and WiFi), communication distance, and connected devices. WiFi consumes a lot of power, and Bluetooth supports up to 8 connected devices in theory. The ability to connect devices is poor. The key is that the device does not have the ability of ad hoc networking, so it must rely on the external network. Zigbee is a bidirectional wireless communication technology based on IEEE 802.15.4 standard (2.4 GHz), low power LAN protocol, and ad hoc low-cost wireless network. It supports up to 65000 devices and has a valuable feature of high-security performance and adopts the AES-128 encryption algorithm [9]. The uses of Zigbee are not as widely promoted as WiFi and Bluetooth, but Zigbee has advantages of low cost, low power consumption (but only for terminal nodes), low complexity, flexible networking (advantages when there are more devices in the network), strong self-recovery ability, small delay, high reliability, and high-security performance.

### 2.2. Zigbee Protocol

#### 2.2.1. Zigbee Networking Mode

**Figure 1:**
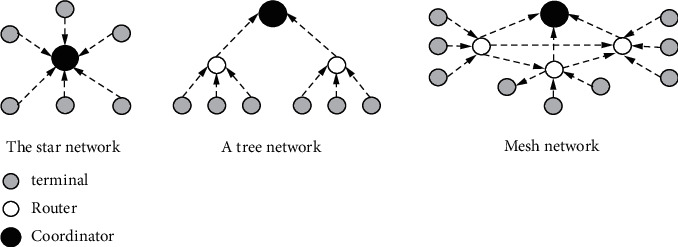
Three network topologies.

The Zigbee protocol specifies three device types for nodes based on their roles in the network: coordinator, router, and terminal. Zigbee technology has strong networking capability and can form three typical self-organizing wireless network types, namely star structure, network structure, and tree structure [10]. As shown in Figure 1:

1. Star network consists of a coordinator (as the central node) and several other terminal nodes, without a routing algorithm. Each node must be within the communication range of the coordinator. The structure has fewer nodes, a simple structure, and low cost. A large amount of data flooding into the coordinator, resulting in network congestion and poor stability. Usually suitable for the network capacity is not high requirements of the environment, such as home automation and other small areas.
2. Tree network is an extension of a star network, adding router nodes. In the tree structure, each router can also have its child nodes. Long-distance transmission can be realized through multilevel hop of router nodes. Terminals cannot connect to their child nodes. Once a routing node in the transmission channel fails, the other nodes of the communication link are disconnected from the network. According to the tree network of the parent-child relationship, the data transmission mode is simple and the transmission path is increased. However, as the scale of the network increases, the transmission delay will become larger and the stability of the network will become worse, which is suitable for the occasion where the timing requirement is not high.
3. Any two router nodes of the mesh network can communicate with each other, and the optimal path selection of the network is completed by the routing table mechanism [11]. It provides multiple routing paths for data packets. If a routing path is damaged (e.g., a module is powered off), the network can choose a new routing path to repair the routing path freely, avoiding data flow collision and blockage, and improving data transmission reliability. Mesh network is suitable for a large number of nodes, so the relative overhead and power consumption is large, suitable for occasions requiring high timeliness and reliability, and is also the most Zigbee network application topology.

Each network topology has its advantages and disadvantages. Considering that the monitoring system has many nodes and large scale, to improve the reliability of data transmission, the network topology is preferred in this system. The mesh network structure does not need to consider the shape of the network when establishing the network and has strong adaptability to the external environment.

Compared with Zigbee 2007, Zigbee 2007 Pro provides greater network support and expands the number of networks. Expanded from 31101 to 65540 [12], it is more suitable for commercial applications and has many enhanced functions, such as alternate addressing, many-to-one routing, and higher security performance [13]. Zigbee 2007 Pro adopts a random address assignment scheme to assign random addresses to newly added devices [14].

Zigbee protocol is different from other network architecture systems, which are divided into 7 layers. That is mainly based on IEEE802.15.4 protocol specification and Zigbee protocol specification. Data transmission specification of NWK layer, APL layer, and APS layer adopts Zigbee protocol specification. The data transmission specification of the physical layer (PHY layer) and Media Access Control layer (MAC layer) adopts IEEE802.15.4 protocol specification, which is Zigbee wireless network [15].

#### 2.2.2. Operation Mechanism of Zstack

To complete data communication between wireless nodes, the whole protocol stack needs to run. The Zstack adds a layer of real-time manipulation called OSAL [16], which operates on top of the OSAL, and the OSAL, a priority-based rotating system, allows for task switching and memory management [17]. The whole system starts from the main function in the ZMain. ZMain.c file in the directory.

The main function is used to initialize the system and perform the multitask polling and query function of the operating system.

*(1) Initialization of the system*. The osal_init_system() function is called to complete the initialization of each module, mainly divided into the initialization of the system clock, initialization stack, initialization of each hardware module, such as I/O, LED, etc., initialization of Flash memory, determine the IEEE64-bit address, initialization of nonvolatile variables, initialization of the operating system, etc. Until osal_start_system (), the function call is the actual operation of the stack.

**Figure 2:**
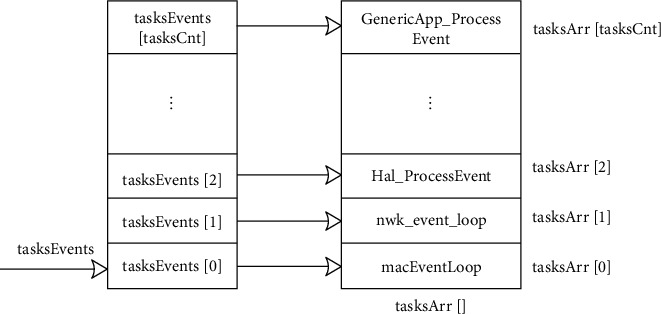
The relationship between the task event table and the task event handler table.

*(2) The execution of the operating system*. After a series of preparations are made for the operation of the operating system after system initialization, the operating system is started. The osal_start_system () function is an infinite loop [18], which continuously detects the occurrence of events and performs corresponding function operations once detected. The operating system needs to do two things: one is to create a task event table. The operating system sets the taskEvents array to hold all the data and adds the event to the task event table. Each item in the array is a pointer to the event handler of each task event table. After processing, it continues to access the event table to see if any events have occurred. TasksArr is set to store the address of each task event handler. When an event occurs, you can find the corresponding task event handler in the function table. Call events = tasksEvents[idx] to retrieve the events to be processed from the task event list. This array contains the sequence number from 0 to tasksCnt. Idx is the sequence number of the priority task (the smaller the value, the higher the priority task). TasksArr [idx] can then be called to perform specific task event handlers depending on the idx in the events= (tasksArr[idx]) (idx, events) statement.  Figure 2 below is a diagram of the task event table and the event handler function table.

### 2.3. Feasibility Requirement Analysis of the System

#### 2.3.1. Analysis of Overall Functional Requirements of the System

**Figure 3:**
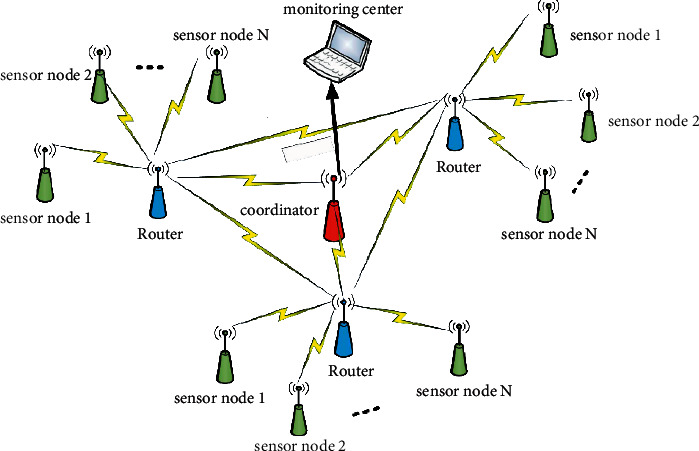
Overall network topology of the system.

Based on the sensor node, router, coordinator, and monitoring center, the ground radiation environment monitoring system is constructed into Zigbee wireless sensor network. Due to the complex geographical location of the environmental area, there are many obstacles and the location of nodes will change, so this design chooses a mesh network structure with multiple data transmission paths to bypass the obstacles by relying on routing relay and migration back to ensure the reliability of transmission and is conducive to centralized supervision distributed in multiple areas. According to each requirement of the monitoring environment of the system, a corresponding sensor node is set.  Figure 3 shows the overall network architecture diagram of the system.

Through the analysis of the overall system workflow, the functional requirements of each part are analyzed as follows:

1. Sensor node function analysis: sensor node is also a terminal node, the node can be configured with one or more sensors, mainly responsible for the acquisition of *γ* radiation dose rate, temperature, humidity, illumination, CO, rainfall, and other environmental parameters, latitude, and longitude coordinates and real-time time information.
2. Router function analysis: mainly responsible for forwarding sensor data collected by nodes in different areas to the coordinator. Relying on the relay function of router uploading and sending, the multi-hop route migration mode is selected to store the collected data and upload it to the coordinator, which makes the whole Zigbee network more extendable and increases the coverage of the network.
3. Function analysis of coordinator: mainly responsible for networking, receiving data, alarm processing, uploading data to an upper computer, and forwarding upper computer commands. Sensor nodes and routers aggregate sensor data to the coordinator. If the data received by the coordinator exceeds the set threshold concentration, the alarm module will be triggered to alarm, and it will be transmitted to the upper computer software of the monitoring center for processing through serial port or Internet, mobile communication network, or system private network.
4. Functional analysis of monitoring center: mainly responsible for displaying environmental data in intuitive decimal numbers and graphs through upper computer software. By reading the encapsulated data directly from the serial port of the coordinator, the environment data are analyzed, processed, and stored according to the communication protocol, and various control operations are carried out according to the analysis results.

#### 2.3.2. System Module Requirement Analysis

Because the monitoring environment is complex and there are multiple interference sources, the following modules are required based on system requirements:

1. Dust sensor: used to detect dust concentration in the environment.
2. Alarm module: used for alarm greater than the danger threshold value; there are two ways to alarm: one is the buzzer and LED sound and light alarm, and the other is GSM SMS remote alarm.
3. GPS positioning module: used to obtain the location and monitoring time of nodes in the environment area. If the monitored data lack geographical location information, the monitoring system will be meaningless. Nodes must have the ability of real-time and geographical location.

## 3. Design of Nuclear Radiation Monitoring System in Floor Survey

Using sensors (photoelectric senssensors information collection devices, design a set of the regional environmental radiation monitoring system, collect the regional environmental radiation doses of radiation, after data encoding, using wireless transmission technology to the control center, control center software after receiving information analysis processing, draw the corresponding curve, As a prerequisite for the controller's next move.

Zigbee network and monitoring host together constitute the regional environmental nuclear radiation monitoring system. The whole system is a hierarchical network structure, with Zigbee sensor nodes at the bottom, Zigbee coordinator and Zigbee router at the middle, and monitoring host at the top [19].

As the high-end equipment of the whole monitoring system, the monitoring host not only needs to monitor the radiation dose data of environmental radiation but also needs to master the health and working status of the sensor node itself [20]. The host not only displays the collected data, but also stores the address of the data source to judge the good or bad situation of the node itself, and timely adjusts the tasks assigned to the node according to the change of the data, as well as prejudges the life cycle of the sensor node. The sensor node itself includes the working conditions of communication components, sensors, residual energy, etc. The remaining energy information of the wireless sensor node determines the working voltage of the node. When the voltage value is low, the reliability of the data transmitted by the sensor node decreases, and the voltage alarm of the monitoring center is triggered. At this time, the battery of the node should be replaced.

### 3.1. Design of Sensor Node

**Figure 4:**
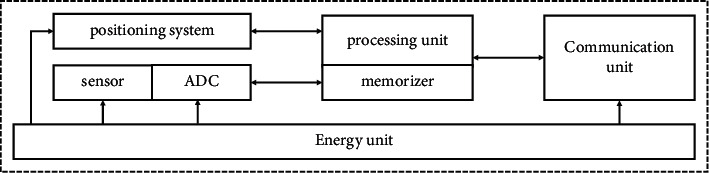
The structure of network node in the system.

The sensor network node structure of the regional environmental nuclear radiation monitoring system is shown in Figure 4.

The structure is composed of an energy unit, Zigbee terminal equipment, Zigbee processing unit, positioning system, and Zigbee communication unit[21]. The Zigbee processing unit is an embedded system with memory, CPU, and operating system. The communication unit includes a serial port direct communication interface and wireless communication module.

### 3.2. System Network Model

When the monitoring environment is not convenient for the host to use on-site for a long time, a base station can be set up close to the monitoring environment. The base station acts as a gateway between Zigbee wireless sensor network and the wired network. Zigbee sensor network first wireless signal to the base station, and the base station through the wired connection will be transmitted to the monitoring host information management module. At the same time, the information management module transmits query and monitoring commands to the base station, which transmits them to the nodes in the Zigbee sensor network.

### 3.3. Distribution of Sensor Nodes

Nodes of the regional environmental nuclear radiation monitoring system based on the Zigbee wireless sensor network adopt a hierarchical structure. The whole network is composed of multiple star networks, which we call clusters. Each star network consists of a central node and multiple terminal nodes. The terminal node is responsible for data collection, and the central node is responsible for data forwarding between nodes. Therefore, the central node of the network (Zigbee router) needs to control the information. The central node can be automatically generated by a clustering algorithm or specified by the monitoring system.

### 3.4. Selection of Evaluation Indexes for Radionuclide Dose

Radiation in the contaminated area formed after regional environmental nuclear radiation is mainly composed of X-rays and rays[22]. The contamination zone itself acts as a radioactive source. Radionuclides (X-rays, rays, etc.) are evaluated by the following criteria:

#### 3.4.1. Activity

Radioactivity is the number of spontaneous decays of radionuclides per unit time, expressed in [Bq]. The definition is as follows:

(1) $$\begin{array}{c} A=\frac{\text{d}N}{\text{d}t}. \end{array}$$

#### 3.4.2. Absorbed Dose

The amount of radiation absorbed by a unit mass of tissue or organ is the absorbed dose, which is measured in *Gy*. One unit of absorbed dose is equivalent to 1 joule of energy absorbed by a unit mass of tissue or organ. The definition is as follows:

(2) $$\begin{array}{c} D=\frac{\text{d}\tilde{\varepsilon }}{\text{d}m}, \end{array}$$

where *D* represents absorbed dose, expressed in grays, 1 *Gy* = 1 J/kg; $\tilde{\varepsilon }$ is the average amount of material absorbed by a tissue or organ per unit mass and is an expected value; and *m* is the mass.

#### 3.4.3. Absorbed Dose Rate

The absorbed dose rate is the amount of radiation absorbed per unit mass of tissue or organ over unit time. The definition is as follows:

(3) $$\begin{array}{c} \dot{D}=\frac{\text{d}D}{\text{d}t}, \end{array}$$

where $\dot{D}$ is the absorbed dose rate in *Gy/s*.

It is difficult to calculate the absorbed dose and absorbed dose rate of radionuclide in the nuclear radiation areas. The absorbed dose of radio nuclear elements in dry air is usually not calculated directly.

#### 3.4.4. Dose Equivalent

Because the total amount of radionuclide absorbed by different people or different organisms is different, the same absorbed dose may not produce the same biological effect. Biological effects are therefore influenced by a variety of factors, such as exposure conditions, types and energies of radiation, individual differences, and dose and dose rate. Considering the above factors, the evaluation index of dose equivalent is introduced. The definition is as follows:

(4) $$\begin{array}{c} H=D\times Q\times N, \end{array}$$

where *H* is dose equivalent in *Sv*, 1 *Sv* = 1 J/kg; *D* is absorbed dose; and *Q* is the quality factor. The quality coefficient of *X* or *v* rays is usually a constant 1, that is, *Q* = 1. *N* represents the product of all other correction factors, usually *N* = 1. So, for *X* or *γ* ray, the focus of this study, the dose equivalent is equivalent to the absorbed dose.

#### 3.4.5. Dose Equivalent Rate

The dose equivalent of a radionuclide element per unit time is the dose equivalent rate. The definition is as follows:

(5) $$\begin{array}{c} \dot{H}=\frac{\text{d}H}{\text{d}t}, \end{array}$$

where *H* is the dose equivalent rate, expressed in *Sv/H*.

It can be seen that dose equivalent and dose equivalent rate more accurately reflect the damage degree of various rays to tissues or organs, while absorbed dose and absorbed dose rate is difficult to be calculated directly and accurately.

### 3.5. Sensors Used by the System

The sensor data acquisition circuit ensures the working performance of the whole system and is mainly responsible for the data acquisition of the entire nuclear radiation environment. Different environmental data require different sensors, and different sensors have different requirements for communication interfaces, so the form of sensor data acquisition circuit is also different [23]. In the selection of sensors, it is necessary to consider both the needs of the nuclear radiation monitoring environment and the interface between sensors and microcontrollers.

Currently, sensors commonly used in the field of environmental monitoring include biosensors, gas sensors, liquid level sensors, thermal sensors, photoelectric sensors, noise sensors, temperature and humidity sensors, etc. According to the different detection indexes, the selected sensor is also different. Considering that the indicators in the field of water quality monitoring include ammonia content, dissolved oxygen, pH value, biochemical oxygen demand, REDOX potential, electricity, etc., optical fiber oxygen sensor and conductivity sensor are usually selected. Given the large number of parameters to be monitored in the field of atmospheric monitoring, there are many types of sensors applicable, including dust particle sensors, sulfur oxide sensors, airborne-burning sensors, etc.

The sensitivity of photocell can be improved by using g photomultiplier tubes because of the high requirement of light particle sensors. The basic working process of the photomultiplier tube is as follows: the photoelectron escapes from the cathode and passes through the cluster electrode for secondary amplification, so the amount of electron escapes from the photocell increases greatly. The Zigbee wireless sensor terminal node designed in this topic selects the abovementioned photoelectric sensor, model FVDK 10N5101, which is used to collect radiation dose in the contaminated area.

### 3.6. Wireless Transmission Technology Route and Analysis

With the development of integration technology, microelectronics technology, and wireless communication technology; the continuous popularization of cheap wireless modules; and the continuous reduction of the installation cost of wireless networking technology, wireless communication module design technology is becoming more and more mature. Mature wireless communication technologies include Bluetooth wireless fidelity, 802.11 b, IrDA, Zigbee, HomeRF, etc.

#### 3.6.1. Track of Bluetooth

Bluetooth technology supports long communication distances and can form a small wireless LAN. A Bluetooth device can establish up to seven simultaneous connections, constantly announce its presence to surrounding devices, and is password protected. The 2.45 GHz channel in the Industrial Scientific Medical (ISM) band is a working channel of the Bluetooth protocol. The transmission distance is 10 m and the transmission rate is 1 Mb/s.

Bluetooth technology can be used in the following areas: as a wireless connection between peripheral devices; realization of data or voice message, information, *n*, and wan connection; and set up personal wireless LAN to realize personal network sharing. However, the chip used in Bluetooth technology is too expensive and has some problems such as information security, short transmission distance, and weak anti-interference ability.

#### 3.6.2. IrDA

The Infrared Data Association (IrDA) is a point-to-point communication technology developed by the Infrared Data Standards Association. The initial transmission rate of 4 Mb/s has reached 16 Mb/s, the initial reception angle was 30°, and now it has reached 120°.

#### 3.6.3. Wireless Fidelity Technology

Wireless Fidel (Wi-Fi) is also a short-range wireless communication technology commonly used in homes and offices. The 2.4 GHz ISM band is also the working band of Wi-Fi. The 802.11 b physical layer defines another data transmission mode in the 2.4 GHz ISM band, and the transmission rate can reach 11 Mbit/s. Although the Wi-Fi technology has the advantages of low threshold, convenient network layout cost, and wide coverage of radio waves, 802.11a and 802.11 b are incompatible with each other, which brings adverse effects on the market promotion.

#### 3.6.4. Zigbee

Zigbee technology is an emerging wireless network communication technology with low power consumption, short distance, and flow rate, the transmission distance can reach 100 meters, and the transmission rate range is 20 Kb/s-250 KB/s. It realizes low speed, short distance, and low cost and is suitable for the regional environmental nuclear radiation monitoring systems.

In the regional environmental nuclear radiation monitoring system, the area contaminated by nuclear radiation is generally large, so the requirements for wireless sensor network are as follows: First, considering that the monitoring of environmental nuclear pollution takes a long time, nodes must save power to ensure low energy consumption. Second, considering that the system does not require high data transmission rate, the cost should be reduced as much as possible after ensuring the required transmission rate. Third, considering the large monitoring range, the number of sensor nodes required is large. It can be seen from the above requirements that the design of a regional environmental nuclear radiation monitoring system based on Zigbee technology will be the best choice.

## 4. System Performance Test and Data Analysis

This chapter mainly uses hardware and software platforms to test the overall function of the wireless network floor survey nuclear radiation environment monitoring system and verify the operation effect of the system design scheme. The main work of the test includes testing the communication of various sensor modules, GPS positioning module, GSM SMS module, and radio-frequency module, and finally realizing data acquisition and transmission.

### 4.1. Node Networking Test

**Figure 5:**
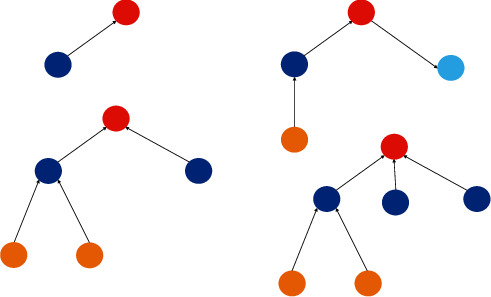
Network topology.

First, five monitoring nodes and one coordinator are prepared to build a wireless ad hoc network, and the topology of the entire Zigbee network ad hoc network is displayed through the Z-Sensor Monitor host computer software of TI Company, as shown in Figure 5:

As can be seen from Figure 5, a network with a mesh topology is successfully formed between nodes. In the figure, two routers (blue) and a sensor node (yellow) are attached to a coordinator (red), among which two sensor nodes are attached to a router. Sensor nodes and routers will calculate the best path to join the network by themselves, so the network structure is flexible and connected everywhere.

### 4.2. Implementation of the Nuclear Radiation Monitoring System

#### 4.2.1. Monitoring System Parameter Setting

Parameters of the nuclear radiation monitoring system based on the Zigbee wireless sensor designed in this article are shown in Tables 2 and 3, whose parameters are set by the JN5121 chip.

#### 4.2.2. Working Mode of Wireless Sensor Network

Zigbee wireless sensor network has two working modes as follows: one supports beacon mode, and the second is the beacon-free mode. In mode 1, after the sensor node is powered on and works normally, the beacon of the sensor network coordinator is first monitored and registered. The coordinator can also set the device's wake mode or sleep mode. Once the data are sent and received, the Zigbee coordinator goes into sleep mode, reducing the energy consumption of the entire sensor network. In mode 2, the Zigbee sensor node is equivalent to an autonomous device that can initiate conversations autonomously. The node only wakes up from sleep mode when an event is triggered. The Zigbee coordinator does not enter sleep mode and needs to continuously listen to external information, so energy consumption is higher than in mode 2.

Considering the convenience of system implementation, the system developed in this article adopts a beacon-free mode to realize data transmission and reception in interrupt mode.

#### 4.2.3. Network Configuration for Wireless Sensors

The Zigbee Coordinator sets the wireless sensor network configuration parameters in the configuration file. The Zigbee Alliance defines standards for configuration files, including how nodes in a network are described and how interfaces to specifications are defined for specific applications.

Considering the convenience of system operation, the system developed in this study configures the network directly in the application program and does not use Zigbee standard configuration file to configure the network parameters. The JZA--boAPPStart () function is used to configure network parameters directly.

#### 4.2.4. Establishment of Wireless Sensor Network

The Zigbee Coordinator is responsible for establishing the entire Zigbee wireless sensor network. The network implementation process is as follows:

First, at the beginning of power-on, the Zigbee Coordinator searches the network for an appropriate transmission channel, which should be the one that is least used or has not been used. Second, a sensor network is established by using the channels selected by the Zigbee coordinator. To facilitate communication identification, a PAN ID is defined for the network. Moreover, once the wireless sensor network is established, Zigbee routers (central nodes) and terminal nodes can be added to the network. After these two types of nodes are added, the Zigbee coordinator assigns appropriate network addresses to them. After the above steps, the nuclear radiation monitoring system based on Zigbee wireless sensor network can be realized.

### 4.3. Commissioning of the Nuclear Radiation Monitoring System

In the process of product development, software and hardware debugging is a key link. In the process of software-hardware debugging, it is possible to simulate various situations that may occur in the field operation, to find and solve problems, and finally improve the performance of the system and achieve the functions that the monitoring system should be able to achieve.

#### 4.3.1. Debug the Hardware Circuit of the System

During debugging, it was found that the program could run on the computer, but not on JN5121. It is considered that there may be a fault in the program download interface circuit. Through continuous checking and debugging, it is finally determined that the program download interface circuit on JN5121 has a pin short connection problem, resulting in the program download failure. Once the short connection problem is resolved, the program can run normally.

The communication between Zigbee wireless sensor network nodes and monitoring hosts was debugged and found that the communication could not be realized. After troubleshooting, the crux of the problem that is rS-485 communication interface circuit failure is found. Since the normal working voltage of the RS-485 communication chip used in this system is +5V, the working voltage provided by the power supply is +3.3 V in the debugging process, resulting in the normal operation of the RS-485 communication interface circuit. After the problem is solved, the RS-485 communication interface circuit runs normally, and the data receiving and receiving between the sensor node and the monitoring host are normal.

Finally, in the operation process of the nuclear radiation monitoring system, the output signal of the sensor node circuit is not ideal, which is greatly affected by external interference, and the output waveform fluctuates greatly and is unstable. After extensive reference, the following conclusions are drawn: the analog circuit is highly sensitive to external disturbance. Therefore, in the combination of the two circuits of hardware, the use of digital and analog ground should not be confused, once confused, will cause high-frequency interference. Usually, there are several ways to separate digital ground and analog ground: for example, to use ohm resistance, inductance, or magnetic beads for separation. This system uses the former separation method, selects 0Q resistance to separate digital ground and analog ground, and adopts inductance to separate digital power and analog power, so that the reliability and anti-interference characteristics of the whole hardware circuit are enhanced, and the output signal waveform becomes stable.

#### 4.3.2. System Software Debugging

When debugging the software program of a nuclear radiation monitoring system based on Zigbee wireless sensor, there is no problem with the basic framework, but there are some programming language problems, which can be solved quickly. For example, in the process of programming the monitoring host data display program, it is found that the display menu has the phenomenon of operation menu dislocation, and the above problem is solved by unifying the signs at the same level.

### 4.4. Performance Test of the Nuclear Radiation Monitoring System

For the built nuclear radiation monitoring system, the network implementation test, data transceiver test, Zigbee coordinator and Zigbee router function test, and stability test were carried out. This study realizes the interface of serial communication in a visual basic environment by writing a serial communication program. One by one, the data receiving situation of the above three nodes are tested. By sending a data ton to check the receiving situation of the corresponding nodes, it is found that the receiving situation of the above three nodes is normal without data loss or garbled characters.

Stability tests are performed in a laboratory environment. The laboratory environment is as follows: the lowest temperature is about 10 °C, the highest temperature is about 25 °C, and the air humidity is about 50%. The nuclear radiation environment monitoring system is placed in the above environment, allowed to continuously for 12 hours, and the stability of the system operation is observed for an emergency. The experimental results show that the regional environmental nuclear radiation monitoring system designed in this study works well and has high stability.

The instrument was placed in the radiation measurement point for field real-time monitoring, and the ambient background and the surrounding environment with ^137^Cs activity were measured respectively. The measurement data are shown in Tables 4, and 5.

By comparing Tables 4 and 5, we can see that: (1) the relative error of environmental dose rate measurement near ^137^Cs source is relatively low; (2) the relative error of dose rate measurement in background environment is high; and (3) through multiple measurements, it is found that the measured value (maximum, minimum, and average values) and relative error are within the fault tolerance range, and the detection accuracy is high, which can be put into practical application.

## 5. Conclusion

Nuclear radiation environmental monitoring has become an important problem of floor exploration. Zigbee wireless sensor network based on deep learning is a powerful tool to realize nuclear radiation environmental monitoring systems. This study adopts Zigbee wireless sensor with JN5121 module as the core to establish a regional environmental nuclear radiation monitoring system and draws the following conclusions:

1. Based on network structure analysis, the sensor terminal node and the central node are arranged manually or in other ways to establish Zigbee wireless sensor network.
2. Zigbee wireless sensor network adopts the JN5121-Z01-M01 chip. The transmission rate of sensor nodes can reach more than 100 Mb/s, and the transmission distance can reach more than 300 m. The data transmission mode is the multi-jump mode. The data collected by the terminal node are transmitted to the central node through this mode, and the central node will transmit the data to the monitoring center after fusion and optimization.
3. CRC Check mechanism and AES-128-bit encryption algorithm are adopted. The safety and reliability of data transmission in the regional environmental nuclear radiation monitoring system is ensured.
4. The regional environmental nuclear radiation monitoring system can not only realize real-time data display but also realize database storage, which can provide historical data for further data analysis.

## Figures and Tables

**Algorithm 1:**
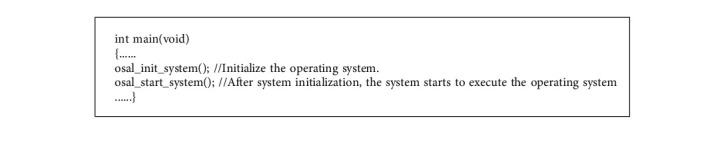
ZMain. C file code in directory.

**Table 1:** Comparison of mainstream wireless sensor networks.

Designation	Zigbee technology	Bluetooth technology	Wi-Fi technology	UWB technology
Cost of the chip	$4/group	$5/group	$20/group	$25/group
Battery life	6–24 months	Several days	Several hours	Several hours
Transmission distance	10–100 m	<10 m	100–300 m	<10 m
Transmission rate	20–250 kbps	1–3 Mbps	11–54 Mbps	1 Gps
Frequency band	868 MHz–2.4 GHz	2.4 GHz	2.4 GHZ	3.1–10.6 GHz
Maximum number of network nodes	65535	8	30	100

**Table 2:** The DC parameters of Zigbee-based wireless sensor network system.

DC character	
Deep sleep current	<1 *μ*A
Sleeping current	<7 *μ*A
Wireless transmission current	50 mA
Wireless receiving current	60 mA

**Table 3:** The RF parameters of Zigbee-based wireless sensor network system.

RF character	
Receive sensitivity	−90 dBm
Sending power	0 dBm
Coverage area	80––100 m
Maximum input signal	−10 dBm

**Table 4:** Background ambient dose rate measurements.

Background surrounding environment	Maximum value	Minimum value	Average	Relative error (%)
1	68.80	67.06	67.52	1.41
2	69.21	68.00	68.53	1.22
3	74.10	71.15	72.23	1.65
4	72.25	68.88	70.06	0.95
5	83.91	80.54	82.12	2.45
6	76.50	74.41	75.72	7.37
7	71.86	68.60	69.78	5.86
8	81.12	78.50	80.00	4.12
9	79.30	75.19	76.55	1.96

**Table 5:** ^137^Cs source environmental dose rate measurements.

Background surrounding environment	Maximum value	Minimum value	Average	Relative error (%)
1	980.13	972.10	978.15	0.32
2	975.28	970.55	972.62	0.76
3	977.61	980.80	973.78	1.32
4	979.56	973.41	975.88	0.87
5	984.91	981.10	983.76	0.21
6	978.52	974.33	976.32	1.43
7	988.78	983.30	985.67	0.64
8	985.21	977.78	979.72	0.82
9	986.57	980.00	982.78	0.75

## Data Availability

The dataset can be accessed upon request.
